# Understanding the complex host response in sepsis: is diabetes the key?

**DOI:** 10.1186/s13054-016-1494-z

**Published:** 2016-10-12

**Authors:** Florian B. Mayr, Sachin Yende

**Affiliations:** 1 CRISMA Center, Department of Critical Care Medicine, University of Pittsburgh, Pittsburgh, USA; 2 Veterans Affairs Pittsburgh Healthcare System, University Drive C, Room 2A128, Pittsburgh, PA 15240 USA

## Main text

Substantial advances have been made in the understanding of the host response to sepsis, but progress in the development of new therapeutic approaches has been disappointing [1]. A potential reason may be the heterogeneity of the host response and the need to precisely match the right therapy to the right patient. Unlike cancer where biomarkers can be analyzed and therapeutic decisions can be made over several days, decisions have to be made within hours in septic patients. Therefore, there is interest in using clinical markers to target therapies instead of using complex biomarker panels. For example, if patients with a particular chronic disease had a unique host response and would benefit from a particular therapy, then the chronic disease could be used to precisely target therapy. There are several reasons to focus on chronic diseases as potential targets. They are common in sepsis patients, they are associated with higher risk of sepsis, and they are often associated with poor outcomes. In particular, studies have focused on diabetes because it is present in approximately 20 % of sepsis patients and is a known risk factor for infection.

Along these lines, van Vught et al. [2] sought to understand if diabetes was associated with a unique pattern of host response in the Molecular Diagnosis and Risk Stratification of Sepsis (MARS) project. They evaluated the immune response in 1104 patients (241 with preexisting diabetes and 863 without) at ICU admission. They used a comprehensive panel of biomarkers to interrogate the host response, a targeted approach (i.e., analyzing 15 plasma biomarkers implicated in the pathogenesis of sepsis) and an unbiased approach by analyzing whole genome expression profiles in leukocytes. They then examined whether preexisting treatment with insulin or metformin had immunomodulatory effects. In their cohort, patients with preexisting diabetes were older and had a higher burden of comorbid conditions, in particular cardiovascular disease, hypertension, and renal insufficiency. Disease severity on ICU admission was similar in both patient groups as reflected by Acute Physiology Scores. Patients with known diabetes were more likely to be admitted with urosepsis (17.4 % in diabetics versus 9.8 % in non-diabetics), which was consistent with a trend towards more Gram-negative infections in diabetics (58.5 versus 51.4 % in non-diabetics). ICU and hospital length of stay, ICU-acquired complications, as well as 90-day mortality were not different between patients with or without preexisting diabetes. Compared to healthy controls, patients admitted with sepsis showed a profound activation of the cytokine (interleukin (IL)-6, IL-8, and IL-10), vascular endothelium (soluble E-selectin, soluble ICAM-1, fractalkine, and angiopoietin-2), and coagulation systems (elevated D-Dimer levels, prolonged prothrombin time and activated partial thromboplastin time) measured up to 4 days after ICU admission. However, none of these markers differed between patients with or without preexisting diabetes. Similarly, neither treatment with insulin nor metformin was associated with differences in clinical outcomes, plasma biomarker levels, or blood leukocyte genomic response after adjusting for baseline differences.

These findings combined with prior results where the host response did not differ in pneumonia patients with and without diabetes that presented to the emergency department [3] suggest that large differences in host response due to diabetes are unlikely to occur. A likely explanation is that the exuberant host response of early sepsis or septic shock overrides any differences due to diabetes. However, it is possible that small differences may still occur. It would be interesting to see whether the proinflammatoy response takes on a different trajectory in diabetic patients after resolution of the hyperacute phase compared to non-diabetics. The lack of influence of insulin and metformin on the acute host response is difficult to interpret as there is no objective measure of medication adherence prior to hospitalization. It is possible that patient’s compliance with home medications decreases in the days leading up to hospitalization for a critical illness like sepsis, which may have attenuated any existing immunomodulatory effect. Finally, given preclinical data that suggest insulin-mediated modulation of local inflammation [4], trying to assess local compartmental inflammation (e.g. lungs or kidneys) may be worthwile pursuing in addition to the overall systemic inflammatory response.

A result that may surprise some is that diabetes was not associated with higher mortality. Indeed, studies examining the association between diabetes and outcomes have shown conflicting results, ranging from higher mortality, no effect, to being protective [3, 5–7]. How do we reconcile these differences? Diabetes could affect outcomes by modifying the risk of developing an infection, increasing the risk of organ dysfunction once infection occurs, and increasing the subsequent mortality. Some studies examining the association between diabetes and outcomes have enrolled patients early during the hospital course, while others have focused on patients with sepsis who are in the ICU. If diabetes merely increases the risk of acquiring an infection and developing organ dysfunction, analyzing patients who are in the ICU alone may underestimate the association between diabetes and outcomes of sepsis. Another reason for the conflicting results could be differences in statistical models with variability in including chronic conditions associated with diabetes and likely to worsen outcomes, such as kidney disease and cardiovascular disease.

In summary, the host response to severe infection is very complex and remains incompletely understood. Chronic conditions, such as diabetes, are unlikely to explain the heterogeneity in the host response to sepsis. Only continuing efforts such as this study by van Vught et al. will allow us to decipher the complex host response and develop precise approaches to target novel therapies.

## Abbreviations

ICU: Intensive Care Unit
IL: Interleukin

## References

[1] Marshall JC (2014). Why have clinical trials in sepsis failed?. Trends Mol Med.

[2] van Vught LA, Scicluna BP, Hoogendijk AJ, Wiewel MA, Klein Klouwenberg PM, Cremer OL (2016). Association of diabetes and diabetes treatment with the host response in critically ill sepsis patients. Crit Care.

[3] Yende S, van der Poll T, Lee M, Huang DT, Newman AB, Kong L (2010). The influence of pre-existing diabetes mellitus on the host immune response and outcome of pneumonia: analysis of two multicentre cohort studies. Thorax.

[4] Filgueiras LR, Capelozzi VL, Martins JO, Jancar S (2014). Sepsis-induced lung inflammation is modulated by insulin. BMC Pulm Med BioMed Central.

[5] Magliano DJ, Harding JL, Cohen K, Huxley RR, Davis WA, Shaw JE (2015). Excess Risk of Dying From Infectious Causes in Those With Type 1 and Type 2 Diabetes. Diabetes Care.

[6] Esper AM, Moss M, Martin GS (2009). The effect of diabetes mellitus on organ dysfunction with sepsis: an epidemiological study. Crit Care BioMed Central.

[7] de Miguel-Yanes JM, Méndez-Bailón M, Jiménez-García R, Hernández-Barrera V, Pérez-Farinós N, López-de-Andrés A (2015). Trends in sepsis incidence and outcomes among people with or without type 2 diabetes mellitus in Spain (2008-2012). Diabetes Res Clin Pract.

